# Increased Activation of Default Mode Network in Early Parkinson’s With Excessive Daytime Sleepiness

**DOI:** 10.3389/fnins.2019.01334

**Published:** 2019-12-12

**Authors:** Leon Qi Rong Ooi, Ming-Ching Wen, Samuel Yong-Ern Ng, Nicole Shuang-Yu Chia, Isabel Hui Min Chew, Weiling Lee, Zheyu Xu, Septian Hartono, Eng King Tan, Ling Ling Chan, Louis Chew-Seng Tan

**Affiliations:** ^1^National Neuroscience Institute, Singapore, Singapore; ^2^Duke-NUS Medical School, Singapore, Singapore; ^3^Singapore General Hospital, Singapore, Singapore

**Keywords:** Parkinson’s disease, resting state fMRI, excessive daytime sleepiness, neural network, independent component analyses

## Abstract

**Background and Objectives:**

The underlying neuropathology of excessive daytime sleepiness (EDS) remains elusive in Parkinson’s disease (PD). We aim to investigate neural network changes that underlie EDS in PD.

**Methods:**

Early PD patients comprising eighty-one patients without EDS (EDS−) and seventeen patients with EDS (EDS+) received a resting state functional MRI scan and the Epworth Sleepiness Scale (ESS). Connectivities within the default mode network (DMN), motor and basal ganglia networks were compared between the EDS+ and EDS− groups. Correlations between network connectivity and the severity of EDS were investigated through linear regression.

**Results:**

EDS+ patients displayed a trend of increased network connectivity of the posterior DMN (pDMN). A significant positive correlation was found between connectivity of the ventromedial prefrontal cortex in the pDMN and ESS.

**Conclusion:**

EDS+ patients are likely to display increased activation in the DMN, suggesting neural compensation in early PD or impaired attentiveness due to mechanisms such as mind-wandering.

## Introduction

Excessive daytime sleepiness (EDS), which refers to the inability to stay awake and alert during the day with sleep occurring at inappropriate times (Zhu et al., 2016) is a ubiquitous non-motor symptom in Parkinson’s disease (PD). Convergent evidence has suggested that EDS is associated with falls, other non-motor symptoms (e.g., depression), and poorer quality of life in PD (Spindler et al., 2013; Chen et al., 2014; Yoo et al., 2019). Understanding the pathogenesis of EDS in PD is important to guide future treatment development.

We have previously demonstrated using resting state functional magnetic resonance imaging (rs-fMRI) decreased activation in the left cerebellum and inferior frontal gyrus, but increased activation in the left paracentral lobule in PD patients with EDS, compared with patients without EDS (Wen et al., 2016). A recent study using diffusion tensor imaging revealed that EDS in PD was associated with decreased microstructural connectivity in the fornices, inferior longitudinal fasciculi and cerebellar peduncles (Ashraf-Ganjouei et al., 2019). To date, there has not been a study examining the intrinsic neural network connectivity that underscores EDS in PD. Specifically, it remains unclear if alterations in the default mode network (DMN) which regulates daydreaming and mind wandering could be associated with EDS in early PD. In this study, we aimed to investigate neural intrinsic functional network features relating to EDS in early PD.

## Methods

### Participants

Early PD patients were recruited from a tertiary referral center. A diagnosis of PD was made by movement disorders neurologists according to National Institute of Neurological Disorders and Stroke (NINDS) diagnostic criteria (Gelb et al., 1999). Additionally, patients were recruited if they met the following criteria: (1) the diagnosis of PD was first made within 2 years before study entry, (2) the absence of other neurological disorders (e.g., stroke and brain injury), (3) Hoehn and Yahr stage ≤ = 3, and (4) no contraindications to magnetic resonance imaging (MRI) scanning.

Patients were divided into two groups: EDS positive (EDS+) group and EDS negative (EDS−) group according to the presence or absence of EDS as defined using the Epworth Sleepiness Scale (ESS). This is a validated, widely used sleep questionnaire that measures the likelihood of an individual falling asleep whilst engaging in eight different activities, with a maximum score of 24. Individuals with ESS scores >10 indicate the presence of EDS. In total, we identified 81 PD patients without EDS (EDS−) and 17 with EDS (EDS+).

This study was approved by the local centralized institutional review board and written consent was obtained from all participating subjects.

### Clinical Assessment

Parkinson’s Disease patients underwent a comprehensive clinical assessment which included motor, cognitive and neuropsychiatric functions upon recruitment. Motor severity was assessed using the Movement Disorder Society (MDS)–Unified PD Rating Scale Part III (MDS-UPDRS-III) (Goetz et al., 2007), cognition was assessed using the Montreal Cognitive Assessment (MoCA) (Dalrymple-Alford et al., 2010), and neuropsychiatric severity was evaluated through the 15-item Geriatric Depression Scale (GDS) (Yesavage et al., 1983), the Hospital Anxiety and Depression Scale (HADS) (Zigmond and Snaith, 1983), Apathy Scale (Starkstein et al., 1992). Finally, the presence of EDS was defined using the Epworth Sleepiness Scale (ESS) (Johns, 1991). This is a validated, widely used sleep questionnaire where the presence of EDS is defined in those with a ESS score of >10, which we partitioned into the EDS+ group (Johns, 1991). The demographic profile of the subjects are presented in Table 1.

**TABLE 1:** Demographical summary of subjects.

	**EDS−**	**EDS+**	***P*-value**
N	81	17	
Gender (M, F)	9,21	14,3	0.073
Age (years)	62.77 (9.85)	65.35 (6.07)	0.324
MoCA	25.99 (2.63)	27.35 (2.69)	**0.018**
GDS	1.59 (1.55)	1.12 (0.99)	0.349
HADS (Depression)	2.25 (2.11)	2.53 (2.15)	0.638
HADS (Anxiety)	2.04 (2.61)	2.29 (2.57)	0.704
Apathy Scale	7.57 (5.70)	9.12 (4.83)	0.205
UPDRS-III	19.75 (10.72)	21.94 (8.86)	0.395
TIV (mm^3^)	1427809 (59272.98)	1418801 (64149.67)	0.351
ESS	4.59 (2.44)	11.62 (1.59)	**<0.001**

*Statistical comparison for gender was done through the chi–square test while the others were compared using the Kruskal Wallis rank–sum test. EDS−, patients without EDS; EDS+, patients with EDS; MoCA, montreal cognitive assessment; GDS, 15-item geriatric depression scale; HADS, hospital anxiety and depression scale; UPDRS-III, movement disorder society (MDS)– Unified Parkinson’s Disease Rating Scale–Part III; ESS, epworth sleepiness scale; TIV, total intracranial volume. The bold values are significant values.*

### MRI Acquisition

Parkinson’s disease patients underwent a brain MRI scan in a 3T Siemens Skyra scanner (Erlangen, Germany), using a 32-channel headcoil. Three sequences were acquired for each patient in their OFF state (medications were withheld at least 12 h before the scan), including:

(1) 3D T1-MPRAGE (Voxel size: 1 × 1 × 1 mm^3^, Field-Of-View: 250 × 250, Slices: 256, TR: 1900 ms, TE: 2.44 ms, TI: 900 ms, Flip angle: 9 degrees).
(2) rs-fMRI (Voxel size: 2.1 × 2.1 × 3.3 mm^3^, Field-Of-View: 200 × 200, Slices: 44, TR: 3000 ms, TE: 30 ms, Repetitions: 150, Flip angle: 90 degrees).
(3) Field map acquisition (Voxel size: 2.1 × 2.1 × 3.3 mm^3^, Field-Of-View: 200 × 200, Slices: 44, TR: 466 ms, TE: 4.92 ms, Averages: 1, Flip angle: 60 degrees).

For the rs-fMRI sequence, in addition to PD medication, patients were instructed not to consume caffeine 12 h before the scan, and to keep awake with their eyes open throughout the scan.

### Image Analysis

The imaging data was analyzed using FSL (Version 5.0). Firstly, the subjects were assessed for motion during the rs-fMRI scan. Subjects were excluded if they either displayed more than 0.3 mm of absolute movement throughout the scan, or had more than 10 frames with 0.3 mm of relative movement between TRs. As a result, seven subjects were removed from the analysis. The structural images of the remaining subjects were preprocessed by denoising of the image using FSL *SUSAN*, non-linear registration of the images to the MNI152 template, and segmented into white matter (WM), gray matter (GM) and cerebrospinal fluid (CSF) maps using FSL *FAST*. Total intracranial volume (TIV) was calculated using FSL *SIENAX*. The functional images were preprocessed by distortion correction using the field map, removal of first 5 TRs for signal stabilization, slice timing correction, motion correction, de-spiking, and regression of nuisance variables arising from CSF, WM and the 6 motion parameters.

The preprocessed data underwent independent component analysis (ICA) in FSL at a set level of 25 components, and the salience, DMN, basal ganglia and motor networks were identified as networks of interested (as illustrated in Supplementary Figure S1). During ICA, the DMN was further split into the anterior and posterior components (the aDMN and pDMN, respectively). The Z scores of correlation for each network was generated by FSL *dual regression*. A mask was generated for each network by thresh-holding the group ICA results at a level of *Z* > 2.5. This mask was used to extract the subject-wise mean Z score for each network.

### Statistical Analysis

The Z scores generated from the dual regression were compared across the two PD groups through FSL *randomize*, which performs a permutation inference for a general linear model. In our exploratory analysis for comparing network differences between the two patient groups. Statistical significance was accepted at a Bonferroni corrected level of less than 0.01 (for the 5 networks of interest).

Univariate linear regression was conducted to determine the relationship between clinical scores (i.e., EDS, GDS, HADS, Apathy Scale and MDS-UPDRS-III scores) and connectivity strength (*z*-scores) for each of the 5 networks for the entire PD cohort in R (version 3.5.4). Statistical significance was set at a Bonferroni corrected level of *p* < 0.01.

## Results

**FIGURE 1:**
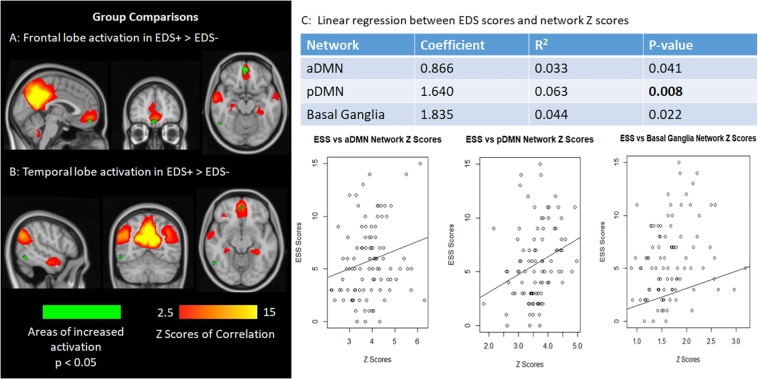
**(A,B)** The frontal lobe (ventromedial prefrontal cortex) and temporal lobe (middle temporal gyrus) activation show increased activation in patients with EDS in relation, although not statistically significant after correction for multiple comparisons. **(C)** Graphs plotting network Z scores against EDS scores for the default mode network and basal ganglia networks.

Although we found no significant differences in the networks of interest in the permutation analysis after correction for multiple comparisons (corrected *p* < 0.01), there was a trend toward significance in the pDMN such that the EDS + group exhibited higher activation as compared to the EDS− group. Specifically, areas in the ventromedial prefrontal cortex and middle temporal gyrus within the pDMN showed increased activation in the EDS + group (0.01 < *p* < 0.05), as illustrated in Figures 1A,B.

However, linear regression analysis showed a significant positive correlation between ESS and network Z-score in the pDMN (*p* = 0.008), after multiple comparison correction. Correlations between ESS and connectivity of the aDMN and basal ganglia networks were marginally significant (0.01 < *p* < 0.05). The associations between ESS and the aforementioned network Z-scores in the networks of interest are shown in Figure 1C. Linear regressions on the remaining networks of interest were not significant.

No correlations were found between the remaining clinical scales and network Z-scores.

## Discussion

As a task-negative network, the DMN has primarily been implicated in PD through disturbed connections arising from dopaminergic loss (Eimeren et al., 2009). We compared early PD patients with and without EDS and showed increased activation of the ventromedial prefrontal cortex within the DMN. Increased activation of the DMN is commonly linked to mind wandering, a state of mind associated with attentional lapses leading to error-prone behavior (Christoff et al., 2009; Kucyi et al., 2016). Although fluctuations in DMN connectivity are not specific to mind-wandering (Kucyi et al., 2016), the attentional deficits linked to this behavior could possibly be associated with the increased prevalence of falls in EDS patients. Changes in activity and atrophy of the temporal region have been found in other studies comparing PD patients with and without EDS (Matsui et al., 2006; Wen et al., 2016), suggestive of possible changes in sensory perception in these patients.

Although no significant association was found between the basal ganglia network and ESS scores after correction for multiple comparisons, increasing network connectivity of the former was seen to have a positive trend of correlation with EDS. PD patients with EDS are commonly thought to show increased dopaminergic loss as compared to those without EDS (Happe et al., 2007; Kato et al., 2012; Yousaf et al., 2018). It is likely that this causes disruptions throughout neuronal signaling in the striatum and consequently, the basal ganglia network and could possibly lead to significant results given more subjects. Notably, positive relationships between EDS and network connectivity found in our study comprising early PD cases could be indicative of a neural compensatory mechanism in the early disease stages. Such a premise, however, would need to be examined using longitudinal cohorts.

Lastly, we found no group differences between the EDS− and EDS + groups with regards to psychiatric measures, nor correlations between the measures and the resting state networks. Although EDS has been linked to psychiatric symptoms such as depression (Xiang et al., 2019), psychosis is rare in the early stages of the disease (Erro et al., 2013), as reflected in this study of early PD patients.

There are several limitations in our study. First and foremost, although EDS was not objectively assessed using the mean sleep latency test, we instead used the ESS, which is a widely used, validated questionnaire that is easily used in research settings. Secondly, the presence of EDS could be due to other previously undiagnosed primary sleep disorders which would require the performance of a video-polysomnography but was not in our study design. Thirdly, it is difficult to standardize the quality of rest of the participants. As PD patients often encounter sleep difficulties (Tandberg et al., 1998; Johns, 2000), it is plausible that the subjects were in differing states of alertness during the MRI scan, affecting the strength of the networks during rs-fmri acquisition. However, extensive control for this would be logistically impractical. Finally, as mentioned previously, this cross-sectional study could be further extended through investigating the longitudinal changes within the EDS + group, compared to the EDS− group.

In conclusion, PD patients in early disease stages with EDS are likely to display increased activation of ventromedial prefrontal cortex in the DMN, suggesting impaired attentiveness due to mechanisms such as mind-wandering or neural compensation. This could potentially be a tool for extending research into the neurological mechanisms that affect PD patients afflicted with EDS.

## Data Availability Statement

The datasets generated for this study are available on request to the corresponding author.

## Ethics Statement

The studies involving human participants were reviewed and approved by the SingHealth Centralised Institutional Review Board, Singapore. The patients/participants provided their written informed consent to participate in this study.

## Author Contributions

LO and M-CW: research project execution, statistical analysis, manuscript preparation, and review. SN, NC, IC, and WL: research project execution. ZX, LC, and LT: research project design, manuscript preparation, and review. SH and ET: research project design and execution.

## Conflict of Interest

The authors declare that the research was conducted in the absence of any commercial or financial relationships that could be construed as a potential conflict of interest.
