# Corrigendum: Increased Activation of Default Mode Network in Early Parkinson's With Excessive Daytime Sleepiness

**DOI:** 10.3389/fnins.2020.00426

**Published:** 2020-05-12

**Authors:** Leon Qi Rong Ooi, Ming-Ching Wen, Samuel Yong-Ern Ng, Nicole Shuang-Yu Chia, Isabel Hui Min Chew, Weiling Lee, Zheyu Xu, Septian Hartono, Eng King Tan, Ling Ling Chan, Louis Chew-Seng Tan

**Affiliations:** ^1^National Neuroscience Institute, Singapore, Singapore; ^2^Duke-NUS Medical School, Singapore, Singapore; ^3^Singapore General Hospital, Singapore, Singapore

**Keywords:** Parkinson's disease, resting state fMRI, excessive daytime sleepiness, neural network, independent component analyses

In the original article, there was a mistake in Table 1 as published. The ages reflected in the second and third columns are inputted incorrectly due to a copy-paste error. The corrected Table 1 appears below.

**Table 1:** Demographical summary of subjects.

	**EDS–**	**EDS+**	***P*-value**
N	81	17	
Gender (M, F)	9, 21	14, 3	0.073
Age (years)	62.77 (9.85)	65.35 (6.07)	0.324
MoCA	25.99 (2.63)	27.35 (2.69)	**0.018**
GDS	1.59 (1.55)	1.12 (0.99)	0.349
HADS (Depression)	2.25 (2.11)	2.53 (2.15)	0.638
HADS (Anxiety)	2.04 (2.61)	2.29 (2.57)	0.704
Apathy Scale	7.57 (5.70)	9.12 (4.83)	0.205
UPDRS-III	19.75 (10.72)	21.94 (8.86)	0.395
TIV (mm^3^)	1427809 (59272.98)	1418801 (64149.67)	0.351
ESS	4.59 (2.44)	11.62 (1.59)	**<0.001**

*Statistical comparison for gender was done through the chi-square test while the others were compared using the Kruskal Wallis rank-sum test. EDS–, patients without EDS; EDS+, patients with EDS; MoCA, montreal cognitive assessment; GDS, 15-item geriatric depression scale; HADS, hospital anxiety and depression scale; UPDRS-III, movement disorder society (MDS)– Unified Parkinson's Disease Rating Scale– Part III; ESS, epworth sleepiness scale; TIV, total intracranial volume. The bold values are significant values*.

The authors apologize for this error and state that this does not change the scientific conclusions of the article in any way. The original article has been updated.

