# Supplementary material for: Increased Activation of Default Mode Network in Early Parkinson’s With Excessive Daytime Sleepiness
Source: Front Neurosci. 2019 Dec 12;13:1334. doi: 10.3389/fnins.2019.01334 (PMC6920242; doi:10.3389/fnins.2019.01334)
Supplement: Supplementary file 1 [file Table_1.DOCX]

**Supplementary Files**


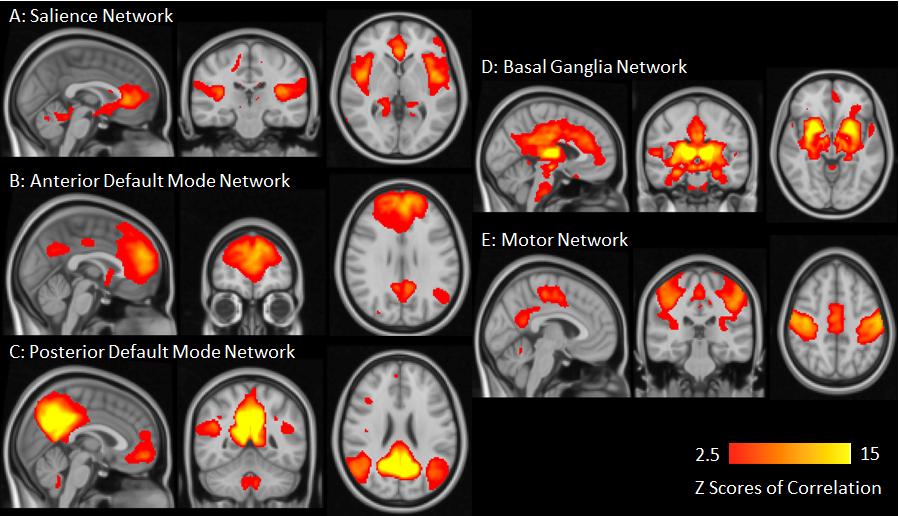


Figure 1: Identification of the salience (A), default mode (B & C), basal ganglia (D) and motor (E) networks through Independent Component Analysis.
